# Emerging negative Atlantic Multidecadal Oscillation index in spite of warm subtropics

**DOI:** 10.1038/s41598-017-11046-x

**Published:** 2017-09-11

**Authors:** Eleanor Frajka-Williams, Claudie Beaulieu, Aurelie Duchez

**Affiliations:** 1 University of Southampton, Ocean and Earth Science, Southampton, SO14 3ZH United Kingdom; 2 National Oceanography Centre Southampton, Southampton, SO14 3ZH United Kingdom

## Abstract

Sea surface temperatures in the northern North Atlantic have shown a marked decrease over the past several years. The sea surface in the subpolar gyre is now as cold as it was during the last cold phase of the Atlantic Multidecadal Oscillation index in the 1990s. This climate index is associated with shifts in hurricane activity, rainfall patterns and intensity, and changes in fish populations. However, unlike the last cold period in the Atlantic, the spatial pattern of sea surface temperature anomalies in the Atlantic is not uniformly cool, but instead has anomalously cold temperatures in the subpolar gyre, warm temperatures in the subtropics and cool anomalies over the tropics. The tripole pattern of anomalies has increased the subpolar to subtropical meridional gradient in SSTs, which are not represented by the AMO index value, but which may lead to increased atmospheric baroclinicity and storminess. Here we show that the recent Atlantic cooling is likely to persist, as predicted by a statistical forecast of subsurface ocean temperatures and consistent with the irreversible nature of watermass changes involved in the recent cooling of the subpolar gyre.

## Introduction

**Figure 1:**
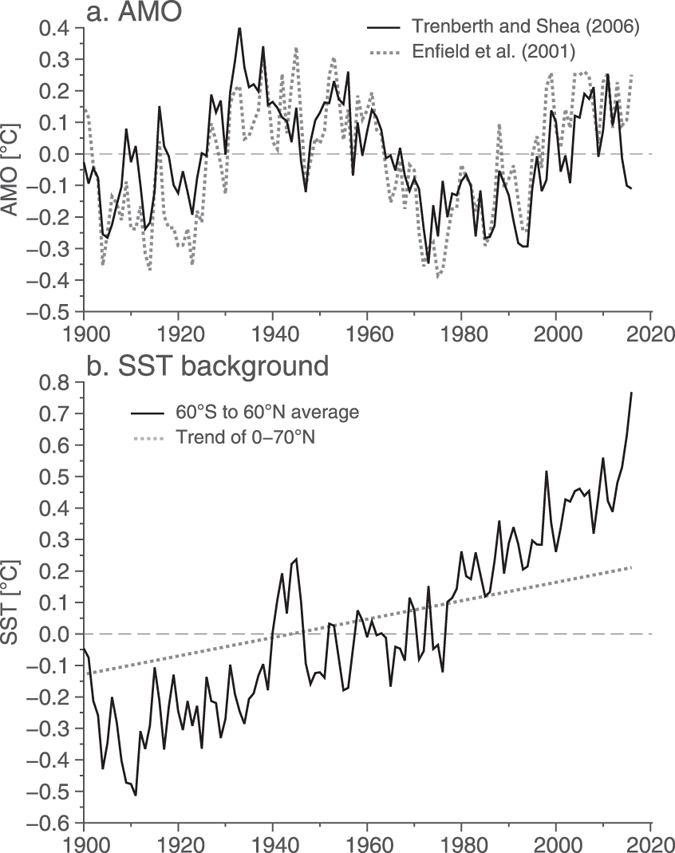
Atlantic sea surface temperature indices and reference field. (**a**) AMO indices following Trenberth and Shea^19^ (black) and Enfield *et al*.^11^ (grey dashed). For the Trenberth and Shea definition, the 60°S–60°N SSTs are subtracted from the North Atlantic SSTs. For the Enfield defintion, the North Atlantic SSTs (0–70°N) are simply detrended. (**b**) The trend that was removed from the North Atlantic SST averages to construct the AMO^11^, and the 60°S–60°N SST that was removed from the 0–60°N, 80–0°W SST^19^.

The Atlantic Multidecadal Oscillation (AMO, Fig. 1a) is an index of decadal variability in the Atlantic based on sea surface temperatures (SSTs). Though the AMO is constructed from SSTs, which respond quickly to atmospheric forcing, the AMO time series is dominated by low frequency–multi-decadal–variations (Fig. 1a). On these timescales, the AMO and Atlantic SSTs co-vary with the strength of the Atlantic meridional overturning circulation (MOC)^1^ where a positive value (anomalously warm Atlantic) of the AMO corresponds to stronger overturning and a negative value (anomalously cool Atlantic) to a weaker MOC. Here, we do not discuss the physical causes of the multidecadal variations in the AMO index, which are debated in the literature^2–7^. Instead, we assess recent tendencies in the AMO index and its relationship to patterns of ocean temperature change.

The last time the AMO was in a cold state was in the 1990s. In the 1990s, the Atlantic shifted from a cold to a warm phase, driven by a strengthening of the MOC and North Atlantic Current^8^ and accompanied by a contraction, warming and slowdown of the subpolar gyre. Over the recent two decades, the AMO has been in a warm phase, corresponding to intensified hurricane activity^9, 10^, decreased rainfall over the US^11^, increased rainfall over India and the Sahel^10^, and a shift in fish stocks in the North Atlantic^12^. A return to a cold phase of the AMO could be accompanied by a reversal of these climate impacts.

Over the recent few years, striking changes in Atlantic SSTs have occurred in the subpolar gyre, where the cold anomaly that developed from 2013–2015 was termed the ‘cold blob’ in the press^13^. This cold anomaly resulted from extremely harsh winters of 2013–2015, characterised by strong surface heat loss^14^ which resulted in persistent cooling of the upper ocean^15^ and drove deep ocean convection^16, 17^. Here, we assess the impact of this cooling on the AMO index, and evaluate the observed changes over the past 3 years relative to the cold AMO period of the 1990s. The AMO index, however, masks any spatial distributions in SST changes, and while the AMO index is negative, the subpolar cold anomaly is accompanied by a warm anomaly in the subtropics. We investigate whether the cold subpolar anomaly is likely to persist and consider how the present cold state of the AMO may evolve.

## Results

### Negative AMO anomaly?

In the recent 3 years, the AMO has become marginally negative, with an average SST anomaly of about −0.1 °C (Fig. 1a). The AMO index reflects variability in the North Atlantic and is typically intended to separate internally forced variations from anthropogenic climate change. Varying definitions of the AMO exist^18^, however, which may highlight different aspects of the basin- or sub-basinscale variability. During the recent reduction of the AMO, both the magnitude and sign of the AMO depend on the definition used to calculate it. The AMO is typically constructed by averaging North Atlantic SSTs and then subtracting a background time series to remove the anthropogenic changes. The Enfield *et al*.^11^ definition removes as the background field a linear trend fit to the Atlantic SST average (Fig. 1b), while the Trenberth and Shea^19^ (hereafter TS06) removes a global SST average as the background field, where the region 60°S–60°N is used to calculate the global temperatures.

In the past 3 years, the global SST average has increased dramatically (Fig. 1b) with the 2015 year being the warmest on record. In contrast, the linear trend used in the Enfield *et al*. definition does not capture nonlinear variations in its background field, so that the background time series shows a steady increase over the recent few years. When this steady increase is removed from the North Atlantic SST average, the values remain positive (a positive AMO index). Due to the inability of the Enfield definition to capture nonlinear changes, and the likelihood of values changing as the trend-endpoints change, we use the TS06 definition here, where the removal of the large global increase in SST from the Atlantic average results in a negative AMO value.

**Figure 2:**
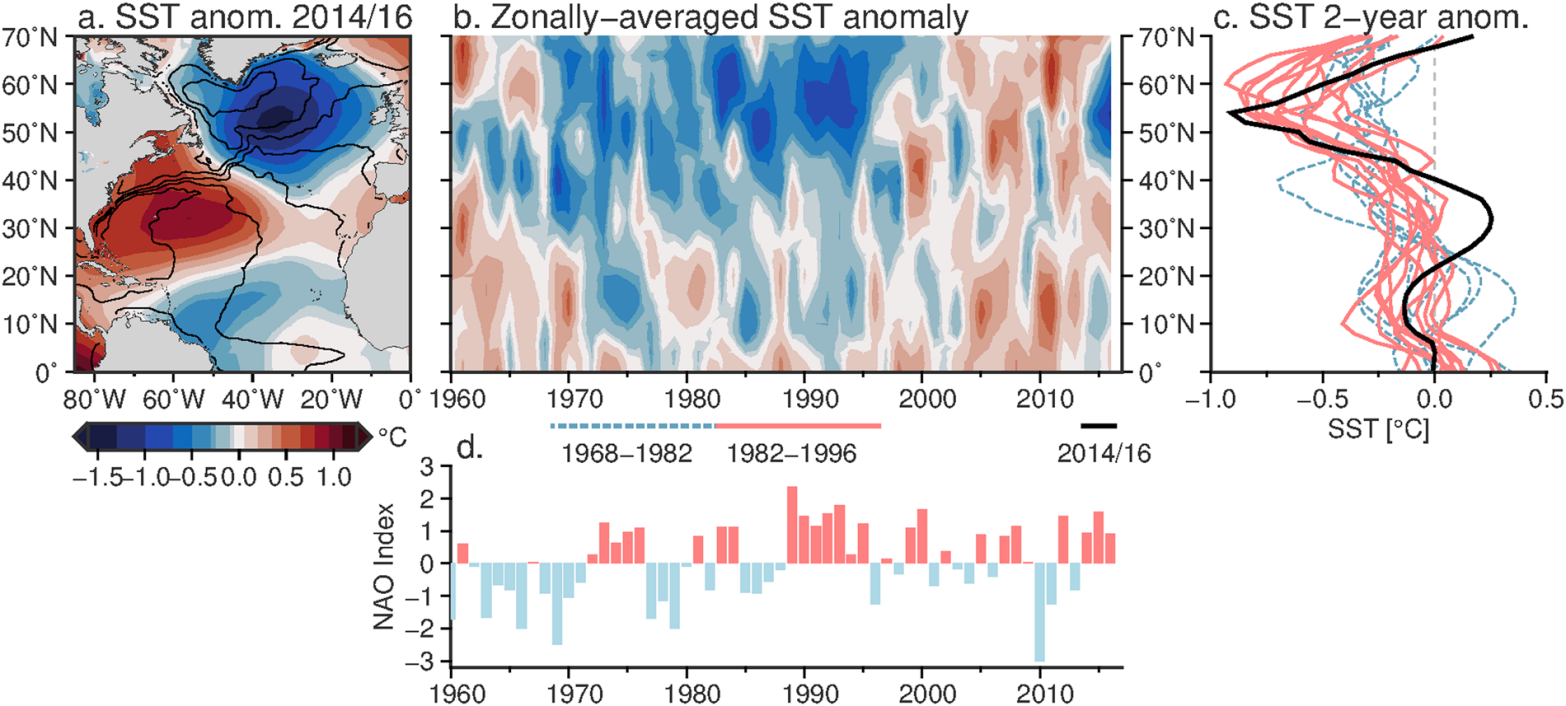
Sea surface temperature anomalies and evolution. (**a**) SST (°C) from the ERSST dataset for the period July 2014–June 2016 are plotted relative to the average over July 2004–June 2012. The map was created using Generic Mapping Toolbox v5.2.1 (gmt.soest.hawaii.edu/). (**b**) Temporal evolution of the zonally-averaged SST as a function of latitude. Both panels show that the recent period has a cold anomaly in the subpolar gyre, but also a warm anomaly in the subtropics. (**c**) Line plots of the zonally-averaged SSTs, where each line represents an average of 2-years. The periods included are the early part of the last cold AMO period (1963–1973, blue) and the late part (1973–1996, red). Black shows the profile for the 2 year period: July 2014–June 2016. (**d**) NAO index where the bar for 2015 represents the December 2015–February 2016 period.

Temperatures are warming globally (Fig. 1b) and also in the Atlantic, but confined to the subtropics. Over the 2014–2016 period, the temperature anomalies show an intensely cold subpolar region, in spite of the relatively warm subtropics (Fig. 2a). The cold anomaly is centred over the eastern subpolar gyre, between Iceland and Cape Farewell (the southern tip of Greenland) at 50°N. In the subtropics, the warm anomaly is concentrated in the western half of the basin around 55°W (Fig. 2a). On longer timescales, the existence of a cooling tendency in the subpolar North Atlantic, while the rest of the Atlantic is warming, has been termed a “warming hole” and is used to diagnose a longer-term slowdown of the MOC^20^. On interannual timescales, an SST tripole (cold subpolar region, warm subtropics, cold tropics) is associated with positive North Atlantic Oscillation forcing^21^. We find that the subpolar cold anomaly is intense enough to swing the entire AMO to a negative index value (depending on the definition used), though the marginal intensity of the negative AMO is due to compensation by the warm subtropics.

To further investigate the time-variability of the meridional pattern of SST anomalies, we consider the evolution of the zonally-averaged SST anomalies. Over the recent 3 years (2013/14, 2014/15 and 2015/16) the cold subpolar gyre has reached the intensity of the anomaly observed in the latter part of the last cold AMO period (early 1990s) (Fig. 2b). In both periods, the subpolar temperature anomalies were intense, while the subtropics were less cold (1990s) or warm (2013–2016). The details of the meridional position and magnitude of the recent cold anomaly are more readily visible in a line plot (Fig. 2c). The line plot highlights a tripole pattern of temperature anomalies, where the magnitude of the subpolar cooling (around 52°N) and subtropical warming (around 31°N) are similar to the late stages of the AMO cold period (1982–1996). A tripole pattern of SST anomalies in the Atlantic is characteristic of SST anomalies under positive NAO conditions and are primarily attributed to anomalous air-sea heat fluxes^21^. While the NAO was positive through several successive winters in both the early 1990s and 2013–2016 (Fig. 2d), the meridional gradient in SST is more pronounced in the 2014/16 period, in association with the warm anomaly in the subtropical gyre.

While the AMO index is marginally negative in the 2014/16 period (compared to more strongly negative in the 1982–1990 s), the AMO index does not capture the spatial pattern of SST anomalies. The persistent meridional gradients in SST over the past few years are likely to influence the atmospheric circulation. The gradients were particularly strong over the North Atlantic Drift region (not shown). In a numerical model, an imposed meridional gradient in SSTs in this region generated variations in sea level pressure, intensifying atmospheric baroclinicity and leading to downstream storminess^22^. In this way, though the patterns of SST anomalies may be set by atmospheric conditions, they can feedback on the atmosphere^23^.

### Changes in Ocean Heat Content

SSTs respond rapidly to atmospheric forcing in addition to subsurface processes and the large scale ocean circulation. The AMO, however, is associated with longer term variations (multi-decadal) in the Atlantic conditions. In this and the next section, we consider evidence for whether the recent changes (cooling in the subpolar regions and warming in the subtropics) and the negative anomaly in the AMO are likely to persist. In contrast with SSTs, subsurface temperature anomalies can persist for months or years, leaving a longer-term impact on the overlying ocean surface, and so here we focus on upper ocean heat content (OHC).

**Figure 3:**
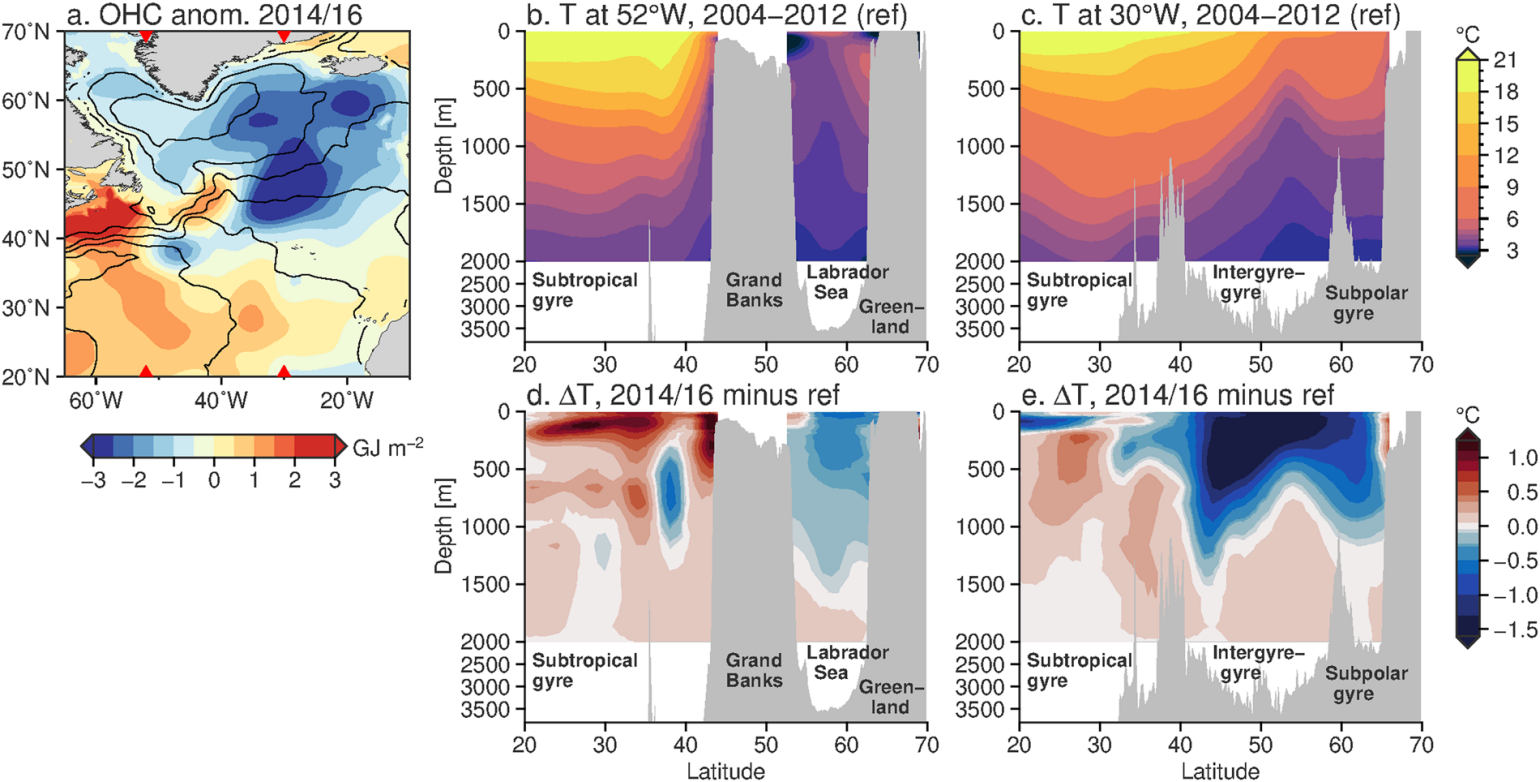
Spatial distribution of ocean temperatures and anomalies. (**a**) Ocean heat content, 0–700 m, for the period July 2014–June 2016 minus the reference period (July 2004–June 2012). Black contours show the mean dynamic ocean topography, while the red triangles indicate the longitudes (52°W and 30°W) for the panels to the right. The map was created using Generic Mapping Toolbox v5.2.1 (gmt.soest.hawaii.edu/). Temperature section from EN4 objectively analysed data at (**b**) 52°W and (**c**) 30°W in the Atlantic, for the reference period. (**d**,**e**) As for (**b**) and (**c**), but showing the difference between the 2014/16 period and the reference period. Positive (negative) values indicate warming (cooling) since the reference period.

The subpolar gyre is typified by cooler water temperatures than the subtropics, with the coldest temperatures in the western subpolar region. The gyre has doming isopycnals, which means that in the centre of the gyre, layers of water are elevated through Ekman transport divergence, supporting the large-scale cyclonic circulation (Fig. 3b,c). The subtropics, in contrast, are significantly warmer, with isotherms tilting strongly in the meridional direction between the subtropics and subpolar gyre around 38°N (Fig. 3b). This strong tilt supports the intense eastward flow of the Gulf Stream. During the 2013/14 winter, the cooling by the atmosphere was strongest over the Irminger Sea^14^ with the subsequent winter further reducing the OHC^15^ (Fig. 3a). A cooling of this magnitude over such a short time scale can be mostly explained by the strong surface heat losses over the combined 2013/14 and 2014/15 winters, with only a secondary role for the weakening of the Atlantic MOC^15^.

During the 2014/15 winter, deep convection penetrated down to 1500 m depth in both the western subpolar gyre (Labrador Sea^17^) and the eastern subpolar gyre (Irminger Sea^16^). Convection to these depths was last observed in the early 1990s^24^. Meridional sections of temperature anomalies from objectively analysed hydrographic observations show the deep penetration of the cold anomaly in the subpolar region (Fig. 3d,e). Remarkably, some of the strongest cooling was observed in the intergyre-gyre region (east of 40°W and around 45°N), over the North Atlantic Current, rather than in the centre of the subpolar gyre. The coldest temperatures were found in the Labrador Sea. In-depth analysis of the temperature changes from 2013–15 found that the cold anomalies were consistent with air-sea flux driven watermass transformation, which is an irreversible process^15^.

In the subtropics the warming is largely confined to the top 300 m in the western half of the subtropical gyre (Fig. 3d). The map further shows some enhanced warming on the North American shelf from Cape Hatteras (around 35°N) to the Grand Banks (around 45°N, Fig. 3a). The subsurface cold anomalies around the latitude of the Gulf Stream (38°N, Fig. 3d) may be associated with a shift in the meridional position of the Gulf Stream, but may also result from the inability of objectively analysed sparse ocean data to capture changes in the sharp temperature gradients in the Gulf Stream region. Unlike changes in the subpolar region, which are typically associated with irreversible, diabatic watermass transformation^25^, the OHC changes in the subtropics are often associated with adiabatic heave (the temporary upwards or downwards movement of water layers) driven by wind anomalies^25, 26^. Heaving water upwards (downwards) through vertical gradients in temperature results in a cold (warm) anomaly at depth. This process can be reversed through a reversal of the wind pattern.

### Persistence of a negative AMO

Here we show that the AMO index tends to covary with OHC anomalies in the subpolar gyre. Due to the expectation that subpolar watermass transformation is an irreversible process, compared to the adiabatic processes in the subtropics, we examine the subpolar OHC for its persistence and potential impact on the lower frequency variations of the AMO index. It is notable that the cold blob disappeared from SSTs during the summer, while the OHC anomaly persisted all year^15^.

**Figure 4:**
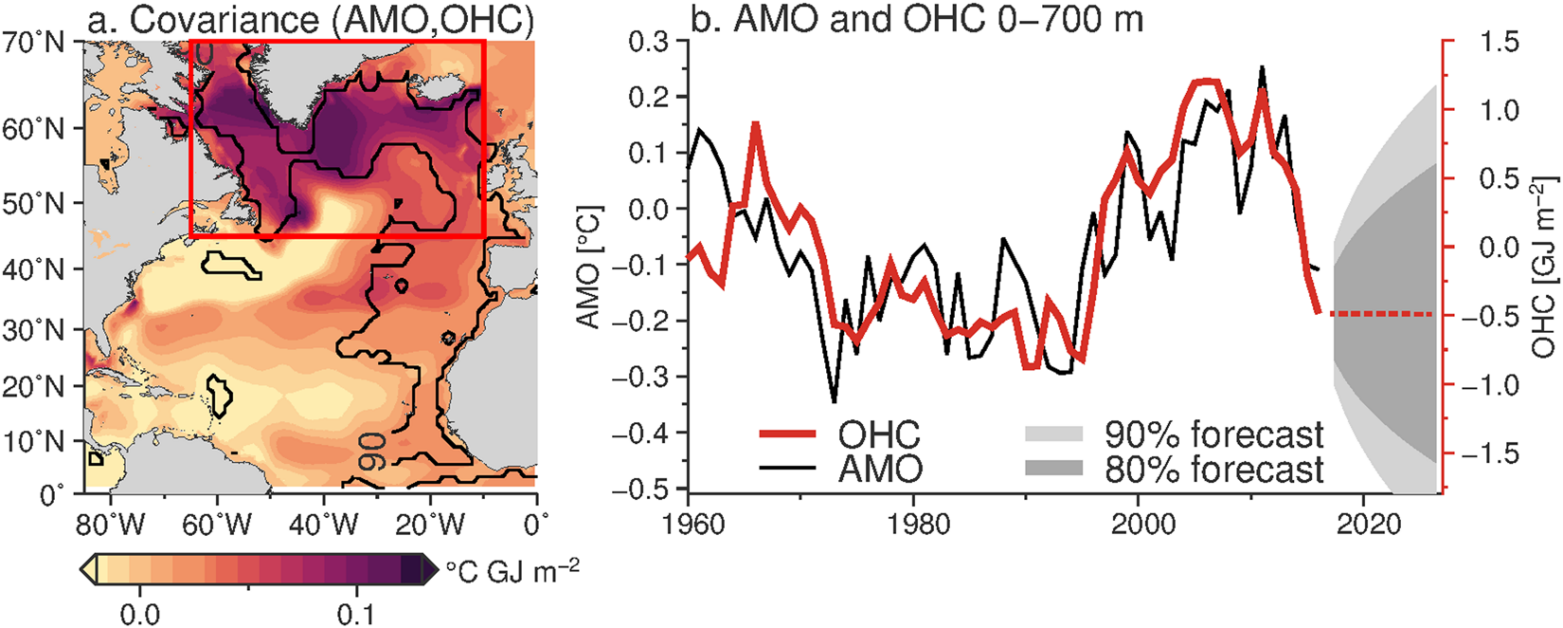
Covariance between OHC and AMO and statistical forecast of subpolar OHC. (**a**) Pointwise covariance between the AMO and OHC over the period 1955–2013 (shading). Significant correlations are contoured at the 90% (black) level, where significance is calculated using the number of degrees of freedom determined by the integral timescale of decorrelation. The red box indicates the subpolar region used in the text. The map was created using Generic Mapping Toolbox v5.2.1 (gmt.soest.hawaii.edu/). (**b**) Time series of the AMO (black) and OHC anomaly (red) from the subpolar North Atlantic (red box in a). The AMO is the Trenberth and Shea^19^ definition, with 0–60°N Atlantic surface temperature minus the 60°S to 60°N average. The OHC anomaly is the NODC 0–700 m average for the subpolar region 45–70°N and 10–65°W.

A pointwise covariance between the AMO (TS06 definition) and upper OHC shows that the relationship between the two is particularly strong in the Labrador Sea (western subpolar gyre) and in the eastern subpolar gyre, north of the North Atlantic Current (Fig. 4, around 40–50°N, 35–45°W). The time series of subpolar OHC (0–700 m, 45–70°N, 10–65°W) tracks the time series of AMO over the recent half-century, including identifying the shift from cold to warm phase in the mid-1990s. In the recent 3 years, the OHC in the subpolar region has reached a low not seen since the 1990s. While we expect OHC anomalies to persist more than SST anomalies^27^ we can also show persistence of OHC anomalies using a statistical forecast. Fitting an autoregressive integrated moving average to the subpolar OHC time series indicates that OHC tends to carry memory from year to year (Fig. 4b). The forecast suggests that the cold anomaly will likely persist over the next 2 years with a probability of 0.8. The persistence of subpolar OHC anomalies, and the dominance of subpolar OHC anomalies on the AMO (Fig. 4a) is consistent with the expectation that OHC, rather than SST, carries the memory of the ocean. In the subpolar region, we further expect that subpolar anomalies are generated by irreversible (diabatic) processes and that once formed, take several years for the anomaly to advect or mix away.

Considering the subpolar OHC changes to be diabatic, we can estimate what forcing would be needed to remove the subpolar cold anomaly over the next few years. An anomaly of −0.5 GJ m^−2^ would require a positive heat flux of 10 W m^−2^ sustained over 2 years to remove, or a northward heat flux anomaly of 0.1 PW sustained over 2 years, or some combination of the above. At 26°N, where the mean northward heat transport is about 1.3 PW^28^, an increase in the northward heat transport of 0.1 PW would equate to a sustained recovery in the overturning transport of 5% over the mean value. These values are not outside the realm of normal variability, but as the overturning transport is presently declining at a rate of 0.5 Sv/yr (3%/year)^29^, the MOC strength would need to recover several Sverdrups of intensity. If the subtropical warm anomaly is a transient feature due to adiabatic heave, it can reduce immediately with a change in the wind stress curl over the Atlantic. If this were to reverse, while the subpolar cold anomaly persists, then the AMO may develop into a more negative state with a basin wide cold anomaly.

## Discussion

The AMO index is designed to capture the multdecadal variability in the Atlantic and is constructed as a large-scale (0–70°N) average of SSTs in the Atlantic, minus a reference time series. However, the choice of reference time series is critical to determining the value of the AMO due to the recent acceleration in global temperature rise. In the recent few years, the magnitude and even the sign of this index can vary depending on the definition used. In one definition, the reference period is the linear trend fit to the Atlantic SSTs^11^, while another uses the globally-averaged SSTs (60°S–60°N)^19^. Globally, the 2015 year was the warmest year on record and represented a sharp uptick in global temperatures. What this means is that when the globally-averaged SST time series is removed from the Atlantic SSTs, the AMO index is negative; a linear trend fit to the Atlantic SSTs is not as strongly positive in the past few years, so when using the Enfield *et al*.^11^ definition, the AMO index is still positive. As the record grows longer, the linear trend may change (or if a linear model is not a good fit to the tendency of the Atlantic SSTs) then the past values of the AMO will change. For this reason, we have used the TS06 definition.

The recent reduction in the AMO index may lead on to further oceanic change. A weak AMO is typically associated with a weak Atlantic MOC in climate models and proxies^20^, and the decreasing trend in the observed Atlantic MOC and meridional heat transport at 26°N^28, 30^ should result in a cooling to the north of 26°N^31^. However, these effects are too slow to explain the rapid cooling that was observed in the subpolar North Atlantic^15^. Instead, we expect that the intense cooling in the subpolar gyre of 2014/16 and intensification of deep convection across the subpolar gyre will lead to an increase in the strength of the Atlantic MOC^1, 32–34^. A direct relationship between convection intensity and the strength of the southward flowing waters of the MOC is elusive in observations^35^, but numerical models suggest that strong convection and their accompanying increases in the density of middepth waters (1000–2500 m) in the Labrador Sea are indeed a precursor to a strengthening of the MOC^32^. In the coming decade, the MOC and associated northward heat transport should intensify, following the increase in subpolar convection^34^), and may reverse the present declining trend of the MOC^29, 30^.

## Conclusions

The AMO index is a convenient measure of the decadal and longer timescale variability in the Atlantic. However, it is a simple proxy built on the large-scale Atlantic SST anomalies and should be used with caution when trying to understand physical processes in the Atlantic or overlying atmosphere. In particular, in the recent few years, the AMO index has become negative, associated with the strong cold anomaly in the subpolar North Atlantic. This cold anomaly is strikingly cold, and reaches deep into the oceans (as seen in ocean heat content), but the magnitude of the AMO index does not reflect the stark cooling that occurred in the past few years. Instead, a warm subtropical anomaly has partially compensated for the cool subpolar regions, resulting in a marginally negative AMO. The direct influence of the oceanic SSTs on the atmosphere will likely differ between the recent cool anomaly and that in the 1980s and 90 s, when the Atlantic had a cool anomaly over much of its expanse (Fig. 2). This is because the atmosphere responds differently to a cool SST anomaly than to an enhanced meridional SST gradient, the latter of which can increase atmospheric baroclinicity and storminess^22^.

While the AMO index is only marginally negative, we use spatial information about the distribution of temperature anomalies (both surface and subsurface) to predict that it will persist. Subpolar OHC variations were driven by anomalous air-sea fluxes^14, 15^ forcing deep convection across the subpolar gyre^16, 36^. Subpolar OHC variations are typically driven by such irreversible processes, which contributes to the persistent nature of OHC variations, as captured by a statistical forecast (Fig. 4). We show that subpolar OHC anomalies in particular are correlated with the AMO, suggesting that the negative AMO will persist. Subtropical changes are typically adiabatic, and reverse with a reversal of the wind-driven heave. Our findings suggest that the negative AMO index will persist and, if the subtropical anomaly reverses, will intensify.

## Methods

Note that an alternate name for the AMO is the AMV (Atlantic Multidecadal Variability) which removes the implicit expectation of an oscillation in the variability. Two time series of the AMO index are used. One was provided by NOAA Earth System Research Laboratory^11^ and derived from monthly 5° resolution Kaplan SST dataset averaged over the Atlantic from 0–70°N and then detrended. The second was calculated from ERSST (Extended Reconstructed Sea Surface Temperature v4) following Trenberth and Shea^19^. According to this defintion, the North Atlantic SST values were averaged, and then the average from them the 60°S–60°N was removed from the North Atlantic time series. For the remainder of the SST calculations, we use the ERSST product since 1854 at 2° resolution.

Ocean heat content (OHC) in the 0–700 m layer was provided by the National Oceanographic Data Center for the period Jan 1950 to Jun 2016^37^. Time series are provided as 3-month averages at 1° resolution. Source data include Argo float profiles, hydrographic data and expendable bathythermograph data as contained in the World Ocean Database. Prior to the Argo period (2004-present), data were relatively sparse, though the North Atlantic remains one of the best sampled regions.

Ocean temperatures for the sections in Fig. 3 are from the UK Met Office EN4 climatology^38^. In the climatology, hydrographic data from ships, probes and Argo float data are gridded at 1° degree spatial resolution and a monthly time interval. Since the Argo array was complete since about 2004, we use a reference period of July 2004–June 2012 in constructing anomalies. Anomalies are typically computed as the data minus the reference period averages, so that a positive anomaly represents a relatively cold area and a negative anomaly represents a relatively warm area.

Data are averaged to annual averages, where the data plotted for 2015 in a time series represents the July 2014–June 2015 average. In the text, the year 2014/15 represents the annual average of monthly values from July 2014–June 2015, while the period 2014/16 represents the 2-year average from July 2014–June 2016. For the NAO index, we used the principal component-based time series from Hurrell (National Center for Atmospheric Research Staff, Hurrell North Atlantic Oscillation Index (PC-Based)). For the NAO, annual values used in Fig. 2 are the December–February averages, so that the value in 2015 represents the average of the value from December 2015–February 2016.

### Relationship between AMO and OHC

A pointwise covariance between the AMO and OHC annual time series over the period 1955–2013 was calculated, where the coefficient of covariance is shown in Fig. 4a. The covariance shows a strong relationship between the OHC and AMO, particularly in the subpolar gyre. The area-weighted subpolar OHC and AMO have a correlation coefficient of 0.7, significant at the 90% level. From this, we conclude that OHC in the subpolar North Atlantic covaries with the AMO, while also having substantial temperature fluctuations.

### Forecasting OHC

**Figure 5:**
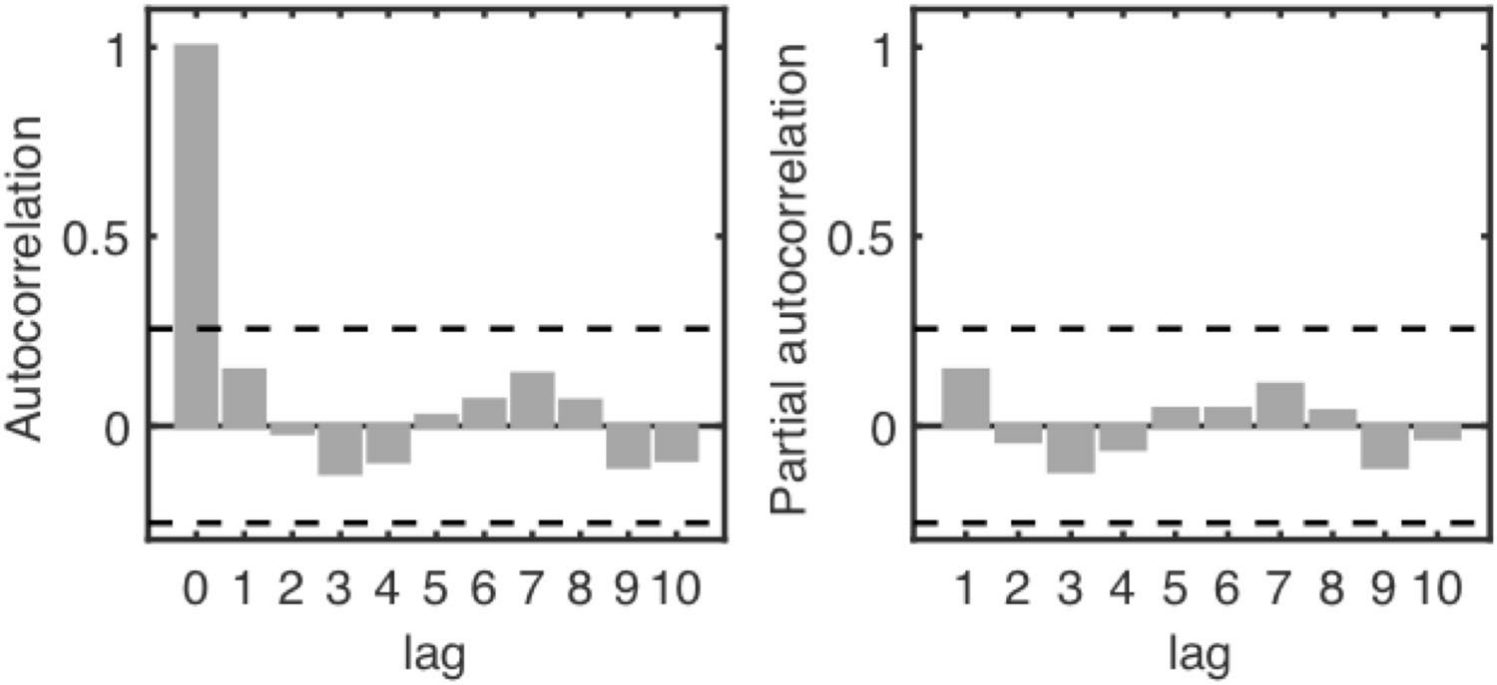
(**a**) Autocorrelation function (grey bars) for the residuals as a function of lag in years. (**b**) Partial autocorrelation function (grey bars). In both (**a**) and (**b**), the black dashed line indicates the 95% confidence interval.

An autoregressive integrated moving average (ARIMA) model was fitted on the subpolar OHC annually averaged values over the region 45–70°N and 10–65°W. The parameters of the ARIMA model were selected using a stepwise procedure fitting all possible ARIMA models from the less complicated ARIMA (0,0,0), which is equivalent to white-noise and does not hold any forecast skills, to an ARIMA(5,2,5). The Akaike information criterion is used to identify the model providing the best likelihood penalized by the number of parameters fitted, thus guards against overfitting. The selected model, an ARIMA(0,1,0), corresponds to a random walk, where each year closely follows the previous year. As measures of model validation, the autocorrelation function and partial autocorrelation function of the residuals are shown in Fig. 5, and indicate that the residuals are independent. Therefore, although very simple the chosen model captures the structure in the OHC time series. To evaluate the forecasting accuracy of the model, we calculate the errors using cross-calibration, i.e. we calculate errors with data that were not used when fitting the model. We use a moving window of ten consecutive observations to fit the model and produce the forecast for the next one. The cross-calibrated root-mean-squared-error (RMSE) is 0.269, showing a high forecast accuracy. As a mean of comparison, the cross-calibrated RMSE for different ARIMA models with the same number of parameters, ARIMA(1,0,0) and ARIMA(0,0,1), are 0.323 and 0.384 respectively. Changing the size of the moving window for the cross-calibration does not have much effect on the accuracy measures. The forecasting was done using the ‘forecast’ R package^39^.

### Data availability

The 0–700 m ocean heat content data are available from the National Oceanographic Data Center (NODC) at https://www.nodc.noaa.gov/OC5/3M_HEAT_CONTENT/. The ERSST sea surface temperature data are available from the NOAA Earth System Research Laboratory (https://www.esrl.noaa.gov/psd/data/gridded/data.noaa.ersst.html). The Met Office EN4 objectively analysed hydrographic data, Gouretski version 4.1.1, are available from http://www.metoffice.gov.uk/hadobs/en4/.
